# Policy options to increase motivation for improving evidence-informed health policy-making in Iran

**DOI:** 10.1186/s12961-021-00737-7

**Published:** 2021-06-07

**Authors:** Haniye Sadat Sajadi, Reza Majdzadeh, Elham Ehsani-Chimeh, Bahareh Yazdizadeh, Sima Nikooee, Ata Pourabbasi, John Lavis

**Affiliations:** 1 Knowledge Utilization Research Center, University Research and Development Center, Tehran University of Medical Sciences, Tehran, Iran; 2 Community-Based Participatory-Research Center, Knowledge Utilization Research Center, and School of Public Health, Tehran University of Medical Sciences, Tehran, Iran; 3 National Institute for Health Research, Tehran University of Medical Sciences, Tehran, Iran; 4 Endocrinology and Metabolism Research Center, Endocrinology and Metabolism Clinical Sciences Institute, Tehran University of Medical Sciences, Tehran, Iran; 5 McMaster Health Forum and Department of Health Research Methods, Evidence and Impact, McMaster University, Hamilton, Canada; 6 Africa Centre for Evidence, University of Johannesburg, Johannesburg, South Africa; 7 Knowledge Utilization Research Center, Tehran University of Medical Sciences, Tehran, Iran

**Keywords:** Evidence-based practice, Evidence-informed policy-making, Policy-making, Motivation, Iran

## Abstract

**Background:**

Current incentive programmes are not sufficient to motivate researchers and policy-makers to use research evidence in policy-making. We conducted a mixed-methods design to identify context-based policy options for strengthening motivations among health researchers and policy-makers to support evidence-informed health policy-making (EIHP) in Iran.

**Methods:**

This study was conducted in 2019 in two phases. In the first phase, we conducted a scoping review to extract interventions implemented or proposed to strengthen motivations to support EIHP. Additionally, we employed a comparative case study design for reviewing the performance evaluation (PE) processes in Iran and other selected countries to determine the current individual and organizational incentives to encourage EIHP. In the second phase, we developed two policy briefs and then convened two policy dialogues, with 12 and 8 key informants, respectively, where the briefs were discussed. Data were analysed using manifest content analysis in order to propose contextualized policy options.

**Results:**

The policy options identified to motivate health researchers and policy-makers to support EIHP in Iran were: revising the criteria of academic PE; designing appropriate incentive programmes for nonacademic researchers; developing an indicator for the evaluation of research impact on policy-making or health outcomes; revising the current policies of scientific journals; revising existing funding mechanisms; presenting the knowledge translation plan when submitting a research proposal, as a mandatory condition; encouraging and supporting mechanisms for increasing interactions between policy-makers and researchers; and revising some administrative processes (e.g. managers and staff PEs; selection, appointment, and changing managers and reward mechanisms).

**Conclusions:**

The current individual or organizational incentives are mainly focused on publications, rather than encouraging researchers and policy-makers to support EIHP. Relying more on incentives that consider the other impacts of research (e.g. impacts on health system and policy, or health outcomes) is recommended. These incentives may encourage individuals and organizations to be more involved in conducting research evidence, resulting in promoting EIHP.

**Trial registration:**

NA.

**Supplementary Information:**

The online version contains supplementary material available at 10.1186/s12961-021-00737-7.

## Background

Putting the most rigorous evidence in the heart of policy development is the key to making health policies/decisions that are not only appropriate but also cost-effective [1–5]. Evidence-informed health policy-making (EIHP) is essential to achieve the Sustainable Development Goals and universal health coverage [6]. EIHP intends to apply the best available evidence (e.g. national context, needs, priorities, and resources) to improve society's health [7–10]. The importance of putting evidence into policy development is widely acknowledged in the literature (e.g. [11–13]). Moreover, it has been emphasized repeatedly in international declarations and statements [14–16].

Despite the importance of EIHP, obtaining and applying high-quality evidence is challenging for many countries and regions [14, 15, 17, 18]. For instance, while the key contribution of research in policy-making has been repeatedly emphasized in the Eastern Mediterranean Region (EMR), health research systems in the region are not yet well developed to generate and use knowledge to improve health and address health inequalities, which in turn will translate into economic development [19, 20].

Tremendous efforts have been made in these countries to create opportunities for linking research to policy (e.g. [12, 21–25]), which resulted in capacity building to enhance the skills and knowledge of policy-makers and researchers; bringing together communities, researchers, policy, and decision-makers; responding to urgent requests for evidence; and changing the health policy-making culture [26]. Furthermore, in response, some advocacy initiatives are designed to facilitate applying evidence in health policies. For instance, in the EMR, “three parallel streams are shaped to promote the use of research evidence in health policies” [23]. These streams are focused on increasing demand among policy-makers for research evidence and valid information, the availability, relevance, and timeliness of evidence, and the structural and process barriers to the use of evidence.

Increased global attention to the use of research evidence in policy-making has motivated Iran’s health system to introduce some initiatives toward promoting EIHP [27, 28]. Because of these actions, the use of research evidence in developing, implementing, and evaluating health policies was more emphasized. However, implementing EIHP has faced some barriers in Iran. Hence, the use of evidence was not well institutionalized in the country's health system [29]. In other words, getting evidence into the policy is not an integral and sustainable part of the national formal system of the country yet [30]. Institutionalizing putting evidence into the development of health policies is challenging due to the complex relations of healthcare organizations (individualized, organizational, and system levels) and the contextual circumstance. Despite this difficulty, this goal must be achieved. In the absence of institutionalization, the success of future initiatives intended to improve the use of evidence will be unclear.

The lack of strong incentives for promoting the use of scientific evidence in making policies has been recognized as a common challenge, particularly in countries where EIHP is in its infancy [14, 15]. For instance, weak motivation to participate in producing research evidence is reported as one of the main barriers to institutionalize EIHP in Iran [31]. As incentives have a key role in motivating individuals to follow certain behaviours [32, 33], incentives can be used to strengthen desires to support undertaking evidence in health policy-making. Thus, as a part of the development of a roadmap for enhancing EIHP in Iran [27], the present study intended to identify effective context-based policy options for strengthening motivations among health policy-makers and researchers to support EIHP in Iran using a mixed-methods design. The evidence provided by the present study can be used by authorities to develop plans intended to motivate individuals to participate in EIHP.

## Methods

The study was conducted in 2019 in two phases, which are described in the following.

### Phase one

We conducted an evidence synthesis through a scoping review and a comparative case study to identify interventions implemented or proposed to strengthen motivations to support EIHP.

#### Scoping review

We systematically searched Scopus and PubMed/Medline databases to identify relevant studies from the time of inception of these databases to 2018. Google Scholar was also mined to increase the comprehensiveness of the search. The search was performed using various combinations of the following keywords: reimbursement, incentive, academic performance, employee performance appraisal, and reward. An example of our search strategy is presented in Additional file 1. The reference list of potentially relevant studies was also scanned. Two authors independently conducted the literature search. Two researchers also independently screened titles and keywords to identify potentially relevant studies, regardless of whether the study has been focused on a specific area. A total of 223 articles (out of 1198) were found to be eligible for review. Then, the two authors independently screened titles and abstracts to identify articles relevant specifically to incentives to support EIHP among researchers and policy-makers. The inclusion criteria were as follows: 1) provided sufficient data about interventions implemented or suggested for strengthening individual or organizational incentives to support EIHP; 2) primary or secondary studies; and 3) published in English. Other types of publication (e.g. commentary, conference proceedings, etc.) and the studies for which full texts were not available were excluded. In the case of a disagreement, a consensus was reached through discussion or, if necessary, a third reviewer was consulted. Data on the following indicators were recorded: year of publication, country area/province of the study, objectives, study design, interventions designed to strengthen incentives and their effectiveness, and administrative considerations if reported. Two authors independently extracted the data using a designed Excel spreadsheet. Disagreements again were resolved by discussion. A narrative approach was employed to synthesize the results of identified studies, using the Push, Pull, Exchange, and Integration model of knowledge translation, proposed by Lavis et al. [34].

#### Comparative case study

A comparative case study approach was used to extract and compare the incentives (either individual or organizational) intended to motivate individuals to support EIHP through an in-depth investigation of a limited number of cases. To this end, first, one country, known for its EIHP, from each continent was selected, except for Antarctica and for North and South America (which were taken together), including the United Kingdom (for Europe), Canada (for the Americas), Australia (for Australia/Oceania), Nigeria (for Africa), and Iran (for Asia). Afterward, from each country, two institutes (one from the pull side and one from the push side) were selected. The selection criteria were being a pioneer organization in terms of EIHP and having an English-language website (Table 1).

**Table 1:** The institutions reviewed to extract the incentives for strengthening EIHP

Country	Name of organization	Type of efforts in EIHP	Website reviewed
United Kingdom	Queen Mary University of London	Push	https://www.qmul.ac.uk/
	Department of Health and Social Care	Pull	https://www.gov.uk/government/organisations/department-of-health-and-social-care
	National Health Service	Pull	https://www.england.nhs.uk/
Canada	McMaster University	Push	https://www.mcmaster.ca/
	Health Canada	Pull	https://www.canada.ca/en/health-canada.html
Australia	Monash University	Push	https://www.monash.edu/
	Australian ministry of health	Pull	www.health.gov.au/
Iran	Tehran University of Medical Sciences	Push	https://www.tums.ac.ir/
	Ministry of Health and Medical Education	Pull	https://behdasht.gov.ir/

The institutions were selected based on experts' opinions. Also, the websites of prominent universities (as push organization), in terms of EIHP, were scrutinized to find documents on motivating people to support EIHP. If the relevant information was not accessible through the websites, a request for information was sent by email. In cases that we could not find relevant information, the university was either replaced (in this case by Queen Mary University of London) or excluded (Ebonyi State University).

In Iran, we selected Tehran University of Medical Sciences (TUMS) (as a high-rank university in research activities) and the Ministry of Health and Medical Education in order to retrieve relevant documents to extract the incentives for supporting EIHP.

Organizational motivation theories have defined motivation as “a positive emotional state resulting from the appraisal of one’s job experiences” [35]. This definition draws attention to two aspects, namely the emotional attachment of employees to their job and supervising their work by the employer. Performance evaluation (PE) processes are common in various organizations, with well-established standards. The results of such processes may provoke employee's emotional reactions [36]. The PE processes often contain two levels: the individual and the organization (sub-organization) [37]. In the present study, individual or organizational PE processes were reviewed to extract incentives designed to support EIHP. Furthermore, given the significant contribution of academic members in providing research evidence, their PE processes, in the form of promotion, were chosen to obtain individual incentives of researchers to support EIHP. For policy-makers, PE processes of macro-level managers and health care staff were selected.

The documents collected from websites of the various organizations were assessed in terms of credibility and adequacy. Then, required data were extracted. Data on the following indicators were recorded: country name, the university/organization’s name, website URL, document’s name, and PE’s dimensions and criteria. The data were compared using a content analysis approach in order to find appropriate interventions to strengthen incentives for supporting EIHP.

At the end of the first phase, two policy briefs were developed, with a special focus on the challenge (i.e. lack of strong incentives (either for researchers or policy-makers) to support EIHP). Afterward, possible options to address the problem were introduced. The origin (scoping review or comparative study), short description, effectiveness, and implementation considerations of each option were described, if possible. The policy briefs were developed to spur the policy dialogues’ participants to be more active [38].

### Phase two

Two separate policy dialogues were held to discuss the challenge, options to address the problem, and key implementation considerations. The following topics were discussed at the policy dialogues: “how to motivate researchers or research institutions to get more involved in producing research evidence” (first dialogue) and “how to persuade policy-makers or policy-making bodies to apply research evidence in their decision-making processes” (second dialogue). These dialogues provided us with an extensive perspective over our research evidence, experiences, and tacit knowledge of those who were involved in EIHP. During these deliberative dialogues, we firstly discussed the issue from different perspectives, and secondly, the impact of various propositions on different groups were examined. Also, a series of discussions were made around various solutions to resolve the problem, along with their feasibility [39]. To ensure the dialogue was based on the most relevant knowledge, the policy briefs prepared in the previous phase were distributed a week before the sessions [40].

Participants of the policy dialogue were selected using a respondent-driven sampling technique. Respondent-driven sampling is a type of snowball sampling used for analysing characteristics of hidden or hard-to-reach populations [41]. We tried to select participants with different backgrounds or experiences (Table 2). Prior to holding the session, by sending an invitation letter the participants were informed about the objectives of the study and the policy briefs. Twelve and eight informants participated in our policy dialogues, respectively, which lasted for 127 and 121 minutes, again respectively. After informing the participants and obtaining their permission, the policy dialogues were audio-recorded. In addition, field notes were also taken to ensure greater accuracy of data collection. The audio files were transcribed verbatim and converted into texts which were reviewed and authenticated by the participants. To ensure anonymity, the results of the analysis were not attributed to participants.

**Table 2:** Characteristics of policy dialogue participants

Participant characteristics	Policy dialogue participants	
	(*N* = 12)	(*N* = 8)
Gender		
Female	5	3
Male	7	5
Position		
Representative from Parliament (pull side)	0	1
MoHME official (pull side)	4	5
Medical university managers (push side)	2	2
Faculty members and researchers (push side)	6	0

*MoHME* Ministry of Health and Medical Education

Data were analysed using the manifest content by two independent authors, in which we described what the informants said, stayed very close to the text, used the words themselves, and described the visible and obvious in the text [42]. Disagreements were resolved by discussion. Coding was conducted using both the inductive and deductive approaches. This method is used in cases where the research topic is well known but little information is available.

Quality criteria explicitly considered in the qualitative data analysis included credibility, dependability, reflexivity, transferability, and confirmability. To ensure credibility, a meeting was held with researchers and principal investigators with a history of performing similar projects to discuss the obtained results. Audit trails were used to ensure dependability. During data gathering and analyses, critical self-reflection about preferences and preconceptions was performed to ensure reflexivity. The transferability of the study was ensured by selecting the appropriate informants to participate in policy dialogues. Confirmability was achieved by obtaining the opinions of a group of participants (member check).

## Results

### Results of the scoping review: interventions intended to increase motivation to support EIHP

**Fig. 1:**
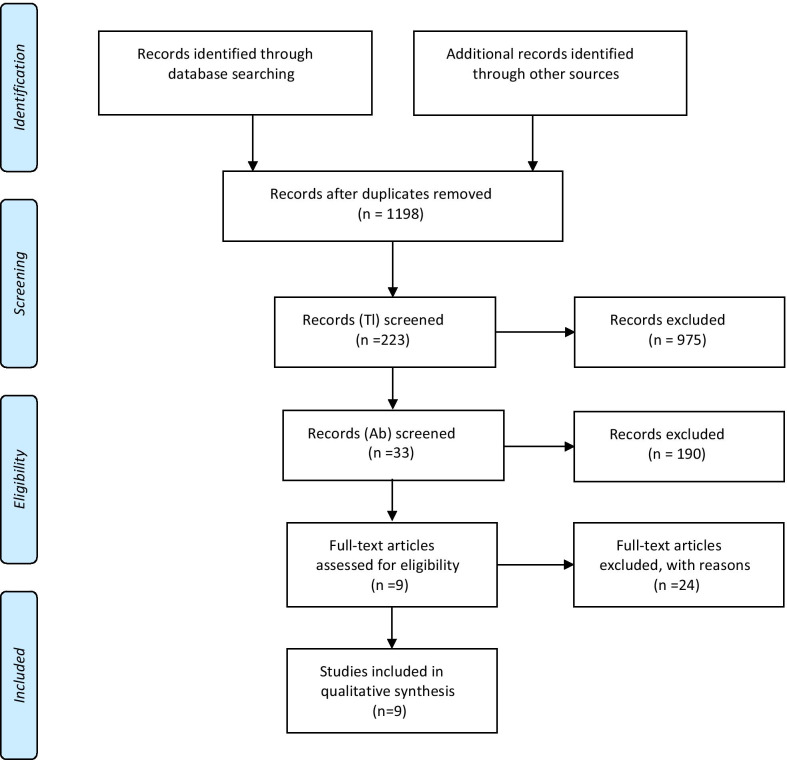
Flow chart of the present scoping review, carried out according to the Preferred Reporting Items for Systematic Reviews and Meta-analyses (PRISMA) guidelines

Thirty-three articles were selected for full-text reading, out of which nine articles were eligible to be included in our evidence synthesis and selected for data extraction (Fig. 1). Our literature review revealed three categories of interventions: (a) push-side interventions, (b) pull-side interventions, and (c) exchange-side interventions (Table 3). The first category contained two parts: interventions related to the PE of academic members [43–46] and interventions related to PE of research institutes and journals [45]. The second category was also classified into two areas of interventions at the individual [22, 46, 47] and organizational [22, 46–48] levels. The third category included (1) new supportive financial mechanisms for health systems research [43, 45] and knowledge translation (KT) activities [45], (2) considering strong incentives (internal or external) for holding dialogues between policy-makers and researchers [44, 48, 49], (3) offering grants based on research impacts [44, 50], (4) conducting KT training courses and encouraging and offering incentives for participating in them [48], and (5) presenting the KT plan when submitting a research proposal, as a mandatory condition [48].

**Table 3:** Interventions identified through scoping review for increasing motivation to support EIHP

Type of efforts in EIHP	Intervention	References
Pushing	Performance evaluation of academic members	
	1. Designing a new career development path for academic members involved in health systems research	[45, 46]
	2. Encouraging the KT activities in forms other than publications	[46]
	3. Revising the performance evaluation criteria of academic members with an emphasis on measuring the impact research on health policy, system, and outcomes	[43, 46, 48]
	Performance evaluation of research institutions and journals	
	4. Designing metrics to measure research impact on policies or health to evaluate the performance of research institutes and periodicals	[45]
	5. Revising the current policies of scientific journals to support health systems research	[45]
Pulling	Individual level	
	6. Job rotation of employee in research institutes	[46]
	7. Using incentives such as sabbaticals or reward for users of evidence	[22]
	8. Putting the measurement of the use of evidence and having the skill of using the evidence in the criteria of employment, retention, performance evaluation and promotion of employees, managers, and organizations	[46, 47]
	Organizational level	
	9. Using the research memorandums in which personal interactions were classified as informal linkages; which personal interactions were classified as informal linkages	[46]
	10. Organizational support (regulation and supportive culture) for using evidence	[22, 47, 48]
	11. Establishing a space for participatory approaches between policy-makers and researchers	[22]
Exchanging	12. Adopting new financial support mechanisms for health systems research and KT activities	[43, 45]
	13. Considering strong incentives to hold dialogues between policy-makers and researchers	[44, 48]
	14. Conducting training courses on KT and encouraging individuals to participate in them	[48]
	15. Required to have a KT plan in research proposals during the submission process	[45]
	16. Proposing performance-based grants	[44, 50]

The effectiveness of the abovementioned interventions has not been evaluated or reported. Only one study, which investigated the impact of two national performance-based grants, reported that one of them yielded positive results. The study also reported that the other programme did not achieve any particular effect [50]. Concerning implementation considerations, only one study reported that changing staff development frameworks is a tremendously difficult task. It had been recommended that these changes should be implemented only on an institution-by-institution basis, with substantial political resolve. Moreover, there should be a gradual approach towards the substitution of traditional staff development frameworks, so that faculty members can perform necessary evaluations using predefined criteria [45].

### Results of the comparative study: incentives for strengthening EIHP in selected countries and Iran

Concerning the individual incentives to support EIHP on the push side, we found that there are several compulsory or optional criteria and areas in the academic promotion process of studied countries that potentially can strengthen the incentives of academics to get them more involved in EIHP initiatives. Nevertheless, there are no compulsory criteria or areas for encouraging academics to be involved in EIHP initiatives or conducting health systems research in Iran. The criteria and areas relevant to EIHP are mainly optional. The most important criteria to judge academic performance in the research area is publications (Table 4). Also, while for most countries the promotion regulations differ from a university to another, a centralized system is dominant in Iran. Furthermore, depending on the discipline and type of membership of the faculty members, different universal assessment criteria are introduced in Iran. Hence, in Iran, the type of membership is the main determinant of expected targets. Concerning organizational incentives, most of the PE criteria are focused on publications in Iran. Only a few of the criteria are concerned with health systems research and research that leads to patents.

**Table 4:** Potential areas and criteria of performance evaluation designed to motivate academics to support EIHP

Institution	Area (related to EIHP)	Criteria (related to EIHP)
Queen Mary University of London	Research	Activities that impact the field Activities essential to further research Output of high-quality, peer-reviewed research publications or other equally recognized forms of research output Significant contribution to the discipline and earned an international reputation
	Engagement with society/impact	Interventions that impact student citizenship positively Involvement in knowledge creation and/or transfer in conjunction with external partner organizations in the industry, commerce, government, or NGOs Activities or interventions that target stakeholders outside academia or address student engagement, widening participation or inequalities within the university Transferred research results to commercial, professional, or other practical use, exploiting these Supported nonacademic stakeholders to engage with research Developing communication strategies to ensure that results of research or outputs or departmental activities or project outputs reach public bodies or the general public Cultivating communication strategies that have led to changes in nonacademic practice/policy and collaboration with partners to improve research topics and practices Identifying new markets for or needed continuing professional development programmes Providing advice to boards of major public bodies on a long-term strategy on a national issue Consulting on policy matters at the national/international level Creating leading research initiatives with nonacademic partners Leading significant business partnerships with major industrial or community partners Providing advice to boards of commercial or public organizations Applying knowledge to improve the performance of public sector organizations Transforming academic outputs, intellectual property, or artworks Showing a significant sustained and externally recognized contribution to student entrepreneurship and enterprise activities Offering a significant record of the transfer of intellectual property into the wider economy including awards for innovation Showing a significant record of responding to the needs and opinions of external groups concerning research topics, processes methodologies, or engagement methods Developing national or international communication strategies to ensure that results of research reach the general public
	Management and collegiality	Providing an identifiable change in a key area of provision or indicator
	Professional practice	Engaging in activities that influence society, economy, government, or public policy Forging links between academia and industry to create opportunities Increasing productivity and efficiency of the healthcare system or other relevant professional and economic fields Directing or providing strategic advice at a national level on the design of clinical or other forms of professional practice to improve the translation of knowledge gained from research activity into the application, resulting in improved patient care, outcomes, or population health, or the equivalent in other professional areas
McMaster University	Research	What would you say is the general quality of the candidate's work? To what degree is the candidate's work original and creative? How significant is it as a scholarly contribution in his or her special area and in the subject more generally? Apart from scholarly work, do you know of any contribution the candidate made to the development of his or her subject in Canada or elsewhere, e.g. through activities in learned societies, organizing conferences, governmental commissions, and so forth? In your opinion, how significant have these activities been?
Monash University	Research (advancing the discipline)	Undertaking impactful research in research, student outcomes, university environment, industry, practice, or community Positive media mentions of a candidate’s research Positive media mentions of team discoveries
	Research (building reputation)	Participating in successful research teams, research units, or centres
	Research (establishing, leading, or participating in research teams)	Positive media mentions of a candidate’s research Positive media mentions of team discoveries
	Research (translation, commercialization, or adoption of discoveries and policy-to-practice by external entities)	Adapting new technologies, patents, designs, and inventions by industry Identifying companies that are on the edge of technology or are utilizing new technologies/patents/designs Changes in government policy or practice resulting from the candidate’s research New legislation, new community funding, and formal reviews of government funding or policy that emerges as a result of the candidate’s research
	Engagement (engagement with industry, government, community, and not-for-profits that contributes to positive economic, social, or cultural outcomes)	Drawing on disciplinary expertise to engage in activities that enhance economic and social outcomes. This may include undertaking voluntary work in legal advice centres or health centres, mentoring of high school science students’ projects, and providing advice/training to community groups on information technology (IT) Effective participation in industry or government advisory committees, that led to a committee report and recommendations that have been well received by the media, government, and/or public Influence on public policy through authoring policy papers or providing evidence to a Royal Commission
TUMS	Research	Generation technical knowledge and inventions

Regarding the incentives to support EIHP on the pull side, in general, we found that in the selected countries, rather than individual and organizational PE, programmes and projects are evaluated. There are particular programmes to evaluate the performance of organizations. It is supposed that progress towards defined projects and achieving defined goals are criteria to evaluate health decision-makers’/managers’ performance. In an ideal situation, since these projects are evidence-based (concerning implementation and evaluation), it can be assumed that the PE process is also evidence-based. Hence, putting evidence into decision-making processes is established in these countries. According to our findings, the selection process of health decision-makers/managers includes reviewing their records and experience. Therefore, instead of appointing managers and evaluating their performance based on translating evidence into practice or policy-making, they are selecting based on earlier records and achieving project goals, where the latter is focused on evidence.

In Iran, however, there are several differences. The current PE and the process of appointment to public bodies contain a series of criteria and areas that can motivate the authorities to apply evidence. However, the current criteria are general and vague, with no definite measure to assess them. Therefore, the assessments are more subjective, rather than precise evaluation. Most criteria are about conducting research and publications, rather than using evidence. The PE process is not properly implemented, and there is no difference between those who use evidence or those who do not. Although the evidence-based practice is strongly recommended to increase efficiency, surprisingly, EIHP has not been institutionalized in Iran. This justifies the need of the country for further support of EIHP.

### Results of the two policy dialogues: policy recommendations to increase motivation to support EIHP in Iran

While a total of 18 policy options were developed, focusing on strengthening incentives to support EIHP in Iran (Table 5), eight were refined through the policy dialogues (with some selected as presented, others merged with other options, and still others rejected). The final eight policy options include the following:

1. Revising the current compulsory criteria and areas of academic promotion in universities to encourage the involvement of academics in performing health systems research, disseminating research results using innovative methods (in addition to research articles), participating in KT activities and bridging the evidence-to-practice gap, and participating more in the peer-review process of health systems research
2. Developing appropriate incentive programmes for nonacademic researchers to motivate them in conducting health policy and systems research (HPSR), disseminating research results using innovative methods (in addition to research articles), participating in KT activities and bridging the evidence-to-practice gap, and participating more in the peer-review process of health systems research
3. Defining criteria to assess the impact of research on the health system, policy, or health outcomes, and using such criteria for evaluating research activities of academic and research institutions and scientific journals as well
4. Revising the current policies of scientific journals to facilitate the submission, peer-review, and publication processes of health systems research in forms other than original articles
5. Revising existing funding mechanisms to support health systems research and KT initiatives
6. Submitting a KT plan as a supplement of research proposals, as an obligatory prerequisite to receive grants
7. Encouraging and supporting different mechanisms for increasing interactions between policy-makers and researchers such as holding policy dialogues, signing and executing memorandums, holding and participating in KT courses, licenses, and documents
8. Revising some administrative processes, including managers and staff PE; selection, appointment, and changing managers and reward mechanisms, to add output-based criteria for EIHP efforts

**Table 5:** Policy recommendations to strengthen incentives to support EIHP in Iran

Policy option	R1*	R2	R3	Final decision	Related quotation
Push side					
Revising the current compulsory criteria and areas of academic promotion with emphasis on measuring the impact of research on health policy, systems, and outcomes	*	*	*	Selected	“We have been saying for several years that there is something wrong with the faculty promotion process. Only the article is important, and in this way, one cannot expect the faculty to do applied research work and to have no advantage over it in the end.” “We are trying to revise the promotion process to make sure that the article is not just a criterion, but accept that it is difficult.”
Developing appropriate incentive programmes for nonacademic member researchers to persuade them to support EIHP			*	Selected	“There is a problem in our country. It may not be in other places that are not among your interventions. Our researchers are not faculty members. There are now many specialized doctoral students who are not necessarily faculty members. Their performance appraisal programme is like that of employees, and that's not true. We need to write a development plan for them on how we want to motivate them to participate in research.”
Encouraging the KT initiatives in forms other than publications	*			Merged	“If we can define which KT activities can be effective, measurable, and evaluated other than the article, these can be added to promotion criteria.”
Designing metrics to measure research impact on policies or health to evaluate the performance of research institutes and journals	*			Selected	“It is ideal to be able to measure the impact of research other than producing an article and evaluate based on it. But it doesn't seem possible.” “You see, now in other countries, the evaluation of the impact of research is done, so this means that it can be done. We should not eliminate an intervention because it is not done in Iran now. We need to let researchers go and work in that field.”
Revising the current policies of scientific journals to support health systems research	*			Selected	“We now have 415 journals. It is completely inappropriate for you to worry that the Persians have a problem with indexing. We currently have 113 of these 415 journals in Scopus that are not English and can be considered for the disseminating of applied research results. Of course, it is debatable what type of manuscript is appropriate for applied research other than the original article.”
Using a variety of media tools for KT initiatives (newspapers, blogs, and social media)	*			Rejected	“Currently, these are in our country, but they are not welcomed because they are not motivated and do not have benefits for individuals.”
Pull side					
Job rotation of employee in research institutes	*			Rejected	“Our human resource regulations allow both faculty and non-faculty to move and mission. But for whom do they do this? What are the advantages of people? Don't be independent at all. These will not be operational until those evaluation criteria change.”
Using incentives such as sabbaticals or reward for users of evidence	*			Merged	“These can be included in the same performance appraisal criteria.”
Putting the measurement of the use of evidence and having the skill of using the evidence in the criteria of employment, retention, performance evaluation, and promotion of employees, managers, and organizations	*	*		Merged	“These can be included in the same performance appraisal criteria.”
Using the research memorandums	*			Merged	“If an institute like NIHR can define and implement a mechanism that, for example, to conduct a required study, the decision is made by both the policy-maker and not necessarily the Ministry of Health, even the parliament, and the scientific community, and speak and state the conditions and expectations. All of this can be seen there.”
Organizational support (regulation and supportive culture) for using evidence	*			Rejected	"It is very general, and that what you said will be done will provide this support and capacity."
Establishing a space for participatory approaches between policy-makers and researchers	*			Merged	“In my opinion, what you wrote is what we have now, but as our colleagues said, it should be motivation, and motivation is through revising our current evaluation system.”
Revising some administrative processes, including manager and staff PE; selection, appointment and changing of managers and reward mechanisms, to add output-based criteria for EIHP efforts			*	Selected	“Nowadays, this system of evaluating the performance of managers and employees, which is central, is of no use. There is no way to differentiate between a manager and an employee who uses evidence. At the level of managers, this is much worse. Now, with this system, how can they find the user manager of the evidence and give him a score? If we want to work in a principled and correct way, as you mentioned in other countries, we must be able to identify with any evaluation system and show the manager who uses the evidence.” “It is imperative to set criteria so that we can objectively measure how much a decision-maker has used the evidence and, of course, not from any evidence, but to find the evidence and evaluate it and take it into account in decision-making.”
Exchange side					
Revision of existing funding mechanisms to support HPSR and KT initiatives	*		*	Selected	“Currently, the share of applied research in research budgets is low. More emphasis is on clinical or basic studies. They [applied research] do not provide immediate results of applied research, but it is not a reason for less attention. If research priorities did not equate applied research with other research, it could motivate. We all know how different and sometimes difficult, the details of applied research are, especially when it comes to engaging with people in the community.”
Encouraging and supporting different mechanisms for increasing interactions between policy-makers and researchers	*		*	Selected	“There is still a gap between practice and science in our system, except in a few cases. We integrated medical education to bring science and practice closer together. But we were not very successful. The heel of Achilles' heel is to be able to use the experiences we previously had as a result of the interaction of action and science and remove this wall.”
Holding training courses on KT and encouraging individual participation	*		*	Merged	“These can be considered in a comprehensive plan for policy-makers and research collaboration.”
Presenting the KT plan when submitting a research proposal, as an obligatory prerequisite to receiving grants	*		*	Selected	“Now, in the proposals of some research centres, it is necessary to submit a KT plan, but only things are filled, even the university site has seen cases for KT activities, but it has not been implemented very well. A bit more serious needs to be taken.”
Proposing performance-based grants	*			Rejected	“Until we fully identify the expected outputs of applied research (other than the paper), it's hard to say what grant's performance is to support it. These are good, but they are more suitable for the next steps.”

*R1: scoping review, R2: comparative case study, R3: policy dialogue

## Discussion

The current study aimed to propose effective context-based recommendations to enhance motivation to support EIHP in Iran, both on push and pull sides.

### Principal findings

Strong incentives can facilitate supporting the EIHP implementation. Some studies mentioned the lack of incentives as a barrier to institutionalize EIHP [14, 15]. Nevertheless, our scoping review disclosed limited evidence on interventions to address these barriers. Moreover, evidence on their effectiveness and implementation considerations were mainly anecdotal and were not based on robust evidence. Future studies are needed to extend our knowledge about the effectiveness of these interventions and to find context-based implementation considerations.

Our findings also revealed that most interventions identified through the review are related to incentives intended to increase interaction between researchers and policy-makers. The evidence-to-practice gap is common in many contexts [51–54]. Thus, most incentives are focused on building strong communications between researchers and policy-makers. Furthermore, the results indicated that the interventions proposed for strengthening incentives among researchers and policy-makers are either individualistic or organizational. As it was mentioned earlier, both individual and organizational incentives are important elements in institutionalizing EIHP efforts [5, 55, 56]. These incentives are considered as a part of a reward system intended to institutionalize desired behaviour(s). Thus, to create or strengthen incentives to support EIHP, individual and organizational goals embedded in the organization's mission and strategic planning should be oriented towards intended behaviours. In addition, reward programmes should also encourage such behaviours.

That is why the mission and vision of academic and research institutions of pioneer countries for implementing EIHP have emphasized creation of incentives for evidence generation and utilization and encouraging interaction between researchers and policy-makers. Following that, the criteria developed for evaluating the performance of individuals and organizations also shifted towards focusing on the extent of research impact on health outcomes, the health system, and policy-making, or collaboration between policy-makers, industry, and community.

Monash University’s strategic plan is a good example, where its mission states that “through excellent research and education, Monash will discover, teach, and collaborate with partners to meet the challenges of the age in service of national and international communities” [57]. After reviewing the strategic plan of Monash University, the following points were extracted: (a) establishing, steering, or participating in successful research teams, units, or centres and fostering interdisciplinary research; (b) translation, commercialization, or contextualization of explorations and conversion of policy into action; and (c) collaboration with the industry, government, and other organizations of the society, including nonprofit organizations, in order to achieve better economic, social, and cultural outcomes [58]. In Iran, not enough attention has been paid to encouraging the EIHP initiatives in academic and research institutions’ vision [59]. More importantly, PE criteria, at both individual and organizational levels, tend to focus on the quantitative evaluation of research impact (number of publications) rather than evaluating the extent of participation or impact of research in health policy-making and systems. The findings highlighted the role of context in spurring health policy-makers and managers to include evidence in their decision-making processes. Pioneer countries have established strong accountability and transparency mechanisms, which avoid ignoring evidence (e.g. embedding personal preferences/interests in the decision-making process). Therefore, the use of evidence cannot be considered as individuals’ or organizations’ PE criteria, whereas, in Iran, lack of strong accountability and transparency mechanisms, which are among the main pillars of health governance arrangements, paved the way for preference-driven decision-making in the absence of evidence [60]. Moreover, insufficient PE criteria have reduced tendencies toward putting evidence into practice [61, 62]. That is why, despite the emphasis laid on EIHP by national policies [63] and recently implemented measures [27], this behaviour has yet not been institutionalized.

### Strengths and limitations of the study

The main strength of the present study is using an evidence-informed approach for proposing solutions to address the identified barriers. To this end, we tried to use global experiences as much as possible to identify interventions and incentives and, at the same time, contextualizing them. Nevertheless, several limitations need to be considered when interpreting the findings, including lack of adequate valid evidence about effective interventions for strengthening the incentives to support EIHP. To remove this limitation, we tried to include all proposed or implemented interventions. Also, we tried to make up for the lack of evidence on effectiveness by providing similar examples from select institutions as the best practices while proposing the recommendations. The second important limitation is restricting the study scope, so that when reviewing the push side, only academic members were considered as researchers. Postgraduate students, independent researchers, experts, and health staff are also potential researchers. Nevertheless, given the diversity and multiplicity of methods available for evaluating their performance and the fact that academic members’ main responsibility is to perform research activities, the study scope was limited to the latter. Furthermore, although there are different types of PE and reward programmes for academic members, in the present study, we only addressed issues related to their promotion, as the most important part of PE and the rewarding process. Moreover, we could not find the required data for other countries’ research institutions; hence, some of them are excluded from the study. When reviewing the pull side, there was no valid data on the method and extent of implementation of PE rules and guidelines and how they have affected managers’ evaluations, which was vague. Compared to other countries, the health system’s plans and projects are defined differently in Iran. In Iran, the process related to research activities is not transparent and, therefore, cannot be evaluated, which adds to the difficulty of evaluating the performance of managers. Furthermore, despite extensive efforts, we could not find criteria that are using by selected countries to evaluate projects or plans. Finally, only internal documents related to the Ministry of Health of Iran and its affiliated organizations were examined, due to their governance role in health policy-making at the macro level; similar documents belonging to nongovernmental organizations were not studied.

### Implications for policy and practice

The authors believe that the proposed recommendations, which were extracted from global experiences and then were contextualized, are useful for creating strong incentives to support EIHP in Iran and other similar contexts, particularly in the EMR. The options mainly focused on revising organizational and individual PE criteria. Any revision should emphasize the impact of evidence on improving general health outcomes, promoting health systems, and the extent of collaboration and interaction between researchers and decision-makers. However, it is a complicated and challenging task. Other recommendations were focused on upgrading certain procedures (e.g. funding and granting paths), which can create greater incentives for conducting health systems research. These options are mainly related to exchange organizations that can be considered in the PE criteria of exchange organizations or embedded in the PE criteria of pushing and pulling organizations.

Several considerations should be considered before implementing any reform, for instance, a pilot programme of revising current incentive systems. Then, if successful, the programme can be scaled up. Given the predicted resistance against any reform measures, stakeholder analysis and advocacy seem necessary. Moreover, there should be monitoring and evaluation programmes for proposed recommendations to determine whether they are effective.

## Conclusions

The current individual or organizational incentive programmes, mainly focused on publications, are not strong enough to motivate researchers to undertake health systems research and encourage policy-makers to get evidence into practice. To motivate health researchers and policy-makers into improving EIHP, it is necessary to rely more on incentives that consider the other impacts of researches (e.g. impacts on health systems and policy, or health outcomes). These incentives may encourage individuals and organizations to participate more in HPSR, which in turn will result in promoting EIHP.

## Supplementary Information

**Additional file 1.** Search Strategy in Pubmed.

## Data Availability

Not applicable.

## Abbreviations

EIHP: Evidence-informed health policy-making
HPSR: Health policy and systems research
KT: Knowledge translation
PE: Performance evaluation
