# Supplementary material for: Policy options to increase motivation for improving evidence-informed health policy-making in Iran
Source: Health Res Policy Syst. 2021 Jun 7;19:91. doi: 10.1186/s12961-021-00737-7 (PMC8186173; doi:10.1186/s12961-021-00737-7)
Supplement: Supplementary file 1 — Additional file 1. Search Strategy in Pubmed. [file 12961_2021_737_MOESM1_ESM.docx]

**Supplementary file 1**

Search Strategy in Pubmed

| Search | Query | Result |
| --- | --- | --- |
| #1 | Decision Making, Organizational [Mesh]  The process by which decisions are made in an institution or other organization. | 10968 |
| #2 | "Policy Making"[Mesh]  The decision process by which individuals, groups or institutions establish policies pertaining to plans, programs or procedures | 23819 |
| #3 | (((((policymak*[Title/Abstract])  OR policy mak*[Title/Abstract])  OR policy-mak*[Title/Abstract])  OR decisionmak*[Title/Abstract])  OR decision mak*[Title/Abstract])  OR decision-mak*[Title/Abstract] | 153980 |
| #4 | #1 OR #2 | 34300 |
| #5 | #4 AND #3 | 5890 |
| #6 | "Evidence-Based Practice"[Mesh]  A way of providing health care that is guided by a thoughtful integration of the best available scientific knowledge with clinical expertise. This approach allows the practitioner to critically assess research data, clinical guidelines, and other information resources in order to correctly identify the clinical problem, apply the most high-quality intervention, and re-evaluate the outcome for future improvement. | 82197 |
| #7 | evidence*[Title/Abstract])  OR informe*[Title/Abstract] | 1727853 |
| #8 | #6 AND #7 | 49669 |
| #9 | #5 AND #8 | 565 |
| #10 | "evidence informed policy making"[Title/Abstract] | 51 |
| #11 | "evidence based policy making"[Title/Abstract] | 136 |
| #12 | #9 OR #10 OR #11 | 689 |
| #13 | #12 Filters: English | 673 |

Search Strategy in Scopus

| #1 | ( TITLE-ABS-KEY ( "policy analys*" ) OR TITLE-ABS-KEY ( "policyanalys*" ) OR TITLE-ABS-KEY ( "policydevelopment*" ) OR TITLE-ABS-KEY ( "policy development*" ) OR TITLE-ABS-KEY ( "policymak*" ) OR TITLE-ABS-KEY ( "policy mak*" ) ) | 173,098 document results |
| --- | --- | --- |
| #2 | ( TITLE-ABS-KEY ( "decision mak*" ) OR TITLE-ABS-KEY ( "decisionmak*" ) ) | 719,386 document results |
| #3 | #1 OR #2 | 873,462 document results |
| #4 | ( TITLE-ABS-KEY ( "Evidence Based Health Care* Management" ) OR TITLE-ABS-KEY ( "Evidence Based Management Health Care*" ) OR TITLE-ABS-KEY ( "Evidence Based Health Care*" ) OR TITLE-ABS-KEY ( "Health Care* Evidence Based" ) OR TITLE-ABS-KEY ( "informe*" ) OR TITLE-ABS-KEY ( "evidence*" ) ) | 3,126,485 document results |
| #5 | #3 AND #4 | 119,959 document results |
| #6 | ( TITLE-ABS-KEY ( "evidence based policy making" ) OR TITLE-ABS-KEY ( "evidence informed policy making" ) ) | 546 document results |
|  | Limit english | 528 document results |
